# Prevalence and Risk Factors for Complications in Patients with Nontransfusion Dependent Alpha- and Beta-Thalassemia

**DOI:** 10.1155/2015/793025

**Published:** 2015-11-18

**Authors:** Poramed Winichakoon, Adisak Tantiworawit, Thanawat Rattanathammethee, Sasinee Hantrakool, Chatree Chai-Adisaksopha, Ekarat Rattarittamrong, Lalita Norasetthada, Pimlak Charoenkwan

**Affiliations:** ^1^Division of Hematology, Department of Internal Medicine, Faculty of Medicine, Chiang Mai University, 110 Intravaroros Road, A. Muang, Chiang Mai 50200, Thailand; ^2^Division of Hematology and Oncology, Department of Pediatrics, Faculty of Medicine, Chiang Mai University, 110 Intravaroros Road, A. Muang, Chiang Mai 50200, Thailand

## Abstract

*Background.* Nontransfusion dependent thalassemia (NTDT) is a milder form of thalassemia that does not require regular transfusion. It is associated with many complications, which differ from that found in transfusion-dependent thalassemia (TDT). Currently available information is mostly derived from beta-NTDT; consequently, more data is needed to describe complications found in the alpha-NTDT form of this disease.* Methods*. We retrospectively reviewed the medical records of NTDT patients from January 2012 to December 2013. Complications related to thalassemia were reviewed and compared.* Results*. One hundred patients included 60 females with a median age of 38 years. The majority (54 patients) had alpha-thalassemia. Overall, 83 patients had one or more complications. The three most common complications were cholelithiasis (35%), abnormal liver function (29%), and extramedullary hematopoiesis (EMH) (25%). EMH, cardiomyopathy, cholelithiasis, and pulmonary hypertension were more commonly seen in beta-thalassemia. Osteoporosis was the only complication that was more common in alpha-thalassemia. The risk factors significantly related to EMH were beta-thalassemia type and hemoglobin < 8 g/dL. The risk factors related to osteoporosis were female gender and age > 40 years. Iron overload (ferritin > 800 ng/mL) was the only risk factor for abnormal liver function.* Conclusion*. The prevalence of alpha-NTDT complications was lower and different from beta-thalassemia.

## 1. Introduction

Thalassemia is a well-known inherited hematologic disorder caused by a decrease or an absence of globin production [1]. Patients with thalassemia suffer from chronic hemolytic anemia and its sequelae. Thalassemia originates from varying genetic abnormalities that result in different clinical presentation. Nontransfusion dependent thalassemia (NTDT) or thalassemia intermedia (TI) is a milder form of thalassemia which does not require regular blood transfusion for survival. This group of thalassemia patients was recognized earlier as a TI but no consensus on diagnostic criteria has been reached due to high clinical variations ranging from asymptomatic to multiorgan involvement [2–9]. The terminology has been changed from TI to NTDT [10]. Generally patients with NTDT can maintain hemoglobin levels at 6–10 g/dL with occasional blood transfusions that may be required with fever, infection, or pregnancy [3, 4, 7, 8, 10]. Complications of NTDT result from chronic hemolysis and tissue hypoxia, causing iron overload and problems in many organ systems [5, 6, 8, 11–20]. According to the largest observational study on thalassemia intermedia (OPTIMAL CARE study; *n* = 584 TI patients), the three most common complications were osteoporosis, extramedullary hematopoiesis (EMH), and hypogonadism, respectively [8].

Several complications that are associated with thalassemia intermedia are less frequently seen in thalassemia major, including EMH, leg ulcers, gallstones, and thrombophilia [8]. One of the most serious complications in NTDT is pulmonary hypertension which can be found in 11–50% of patients and leads to heart failure; the most common cause of death in NTDT patients [3, 4, 6, 8, 11, 13, 14, 16].

In our region, the proportion of patients classified by thalassemia type is changing due to advances in prenatal diagnoses and early detection. Higher numbers of NTDT patients are diagnosed and more fetuses with severe thalassemia are terminated.

Many previous studies aim to establish predictive factors for thalassemia complications and report that mechanisms for complications in thalassemia are multifactorial [3, 6, 8, 12, 15, 21–27].

In our region, the prevalence of alpha-thalassemia is greater than that of beta-thalassemia which is different from the prevalence found in other regions [25, 26, 28–30]. The lack of studies and clear guidelines in this group can present a significant clinical challenge. This study aims to elucidate the prevalence of complications and identify predictive factors affecting complication of both alpha- and beta-NTDT patients.

## 2. Material and Method

We retrospectively reviewed medical records of NTDT patients who attended the Chiang Mai University Hospital Adult Hematology Clinic for the two-year period from January 1, 2012, to December 31, 2013.

### 2.1. Population

The NTDT patients, age 15 years or older, were included in the study. NTDT is defined by thalassemia disease that does not require regular transfusion for survival [10]. However, the definition of transfusion varies among studies. We used the criteria of less than an average of three transfusions per year for the purpose of the study. The patient needed to visit the clinic at least once in order to be enrolled.

The diagnosis for the type of NTDT patients was made by hemoglobin analysis using a high-performance liquid column chromatography (HPLC) method. The molecular confirmation of *α* ^0^-thalassemia (Southeast Asian or Thai deletion) and HbCS was done for cases with HbH disease and HbH with Hb Constant Spring (HbH/CS) disease. Molecular diagnosis of beta-globin mutations was done in cases with beta NTDT when the results from hemoglobin analysis by HPLC method showed abnormal hemoglobin peak other than Hb E.

### 2.2. Data Collection

From January 1, 2012, to December 31, 2013, medical records of NTDT patients who met the inclusion criteria were reviewed. Data collected from medical records included demographic characteristics and diagnosis obtained by hemoglobin analysis. Also, findings from physical examination, laboratory investigations, and records of complications were recorded. The definition of conditions and complications in this study are shown in Table 1 [8].

### 2.3. Complications

Complications of NTDT patients were retrospectively collected from the medical record.

All NTDT patients had regular evaluation and investigations for these complications: three-monthly liver function tests and serum ferritin, annual tests for endocrine function which included fasting plasma glucose, thyroid function test, and hormonal assays for hypogonadism. Hepatitis B and C virus test were also done annually. Chest radiograph and echocardiogram were obtained for suspected cases of cardiomyopathy. Spine radiograph and bone mass densitometry were conducted in suspected osteoporosis cases.

For other complications such as extramedullary hematopoiesis (EMH), pulmonary hypertension (PHT), thrombosis, cardiomyopathy, cholelithiasis, pseudoxanthoma elasticum (PXE), leg ulcers, and osteoporosis (OP), the information was obtained retrospectively from medical records. Investigations for complications listed in Table 1 were conducted for putative cases where risk factors were present.

### 2.4. Statistical Analysis

Data were entered into database, crossed-checked, and analyzed using SPSS statistics software. Descriptive results of categorical and continuous variables were expressed as mean (±SD) or median (range in continuous variables) depending on their distribution or as percentages of the group from which they were derived (categorical variables). The Chi-square test or Fisher exact test was used to compare categorical variables and Student's *t*-test was used to compare between continuous variables as appropriate. Variables that were significantly related to complications or with *p* values less than 0.05 in the univariate analysis were entered into the multivariate analyses. Multivariate logistic regression analysis was used to identify independent risk factors for complications. Odds ratios (OR) and 95% confidence intervals (CI) were calculated for all associations that emerged. A *p* value less than 0.05 was considered as statistically significant.

## 3. Results

### 3.1. Patient Characteristics

During the study period, 250 thalassemia patients attended our clinic. Of these, 100 NTDT patients who matched our inclusion criteria were included in this study, 60 patients (60%) were female.  Table 2 summarized patient demographics, underlying diseases and conditions, and clinical characteristics. The median age was 38 years (range 19–78 years). More than half of patients (54%) were diagnosed with alpha-thalassemia. The mean ferritin level was 1,563.46 ng/mL while 76% and 44% of patients had ferritin levels more than 800 and 2,500 ng/mL, respectively. Chronic hepatitis B infection (27%) was the most common comorbid condition.

### 3.2. Complications and Treatment Outcomes

**Figure 1:**
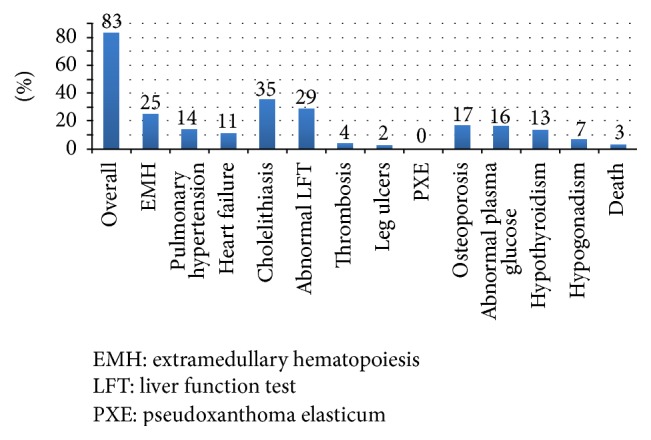
Percentage of complications and mortality in study population.

Figure 1 summarizes patient treatments and the outcomes. Fifty-five of 100 patients (55%) received iron chelation treatment for iron overload, and 33 of these patients (33%) underwent a splenectomy. Overall, complications occurred in 83% of the study population. The three most common complications were cholelithiasis (35%), abnormal liver function (29%), and EMH (25%). Other complications included osteoporosis (17%), abnormal plasma glucose (16%), pulmonary hypertension (14%), hypothyroidism (13%), cardiomyopathy (11%), thrombosis (4%), hypogonadism (7%), and leg ulcers (2%), respectively.

The radiologic investigations including plain film, CT scan, and MRI were used to detect EMH complications. Paravertebral soft tissue masses were found in nine patients, thalassemic bone change was found in five patients, and 11 patients had both paravertebral soft tissue and thalassemic bone change. All paravertebral soft tissue masses (20 patients) were found in the thoracic spine region. Thalassemic bone changes were found in the ribs (14 patients), femur (one patient), and spine (one patient).

### 3.3. Complications in Alpha and Beta-NTDT

The differences of complications classified by type of thalassemia were summarized in Table 3. The most common complications were similar between alpha- and beta-thalassemia groups: cholelithiasis and abnormal liver function test. However, the prevalence of cardiomyopathy, cholelithiasis, and pulmonary hypertension was higher in beta-thalassemia but the differences were not statistically significant. Osteoporosis was the only complication that was more commonly seen in alpha-thalassemia.

Though not statically significant, beta-thalassemia patients tended to have higher clinical severity and required further treatment more frequently than those with alpha-thalassemia. The mean ferritin level for the beta-thalassemia group (1,971 ng/mL) was higher than the alpha-thalassemia group (1,202 ng/mL). Seventy-four percent of beta-thalassemia patients received iron chelation as compared to 39% in alpha-thalassemia patients. Splenectomy was performed in 54.3% of beta-thalassemia and only 14.8% of alpha-thalassemia patients.

### 3.4. Risk Factors Affecting Complications

Results from the univariate analysis of significant risk factors for each complication were shown in Table 4. The following factors were significant in the model: extramedullary hematopoiesis, female gender (*p* = 0.05), beta-thalassemia (*p* = 0.031), hemoglobin level below 8 g/dL (*p* = 0.003), platelets above 400,000/mm^3^ (*p* = 0.025), maximum ferritin more than 800 ng/mL (*p* = 0.004), iron chelation (*p* = 0.001), and splenectomy (*p* = 0.019). Splenectomy was also associated with heart failure (*p* = 0.035) and hypogonadism (*p* = 0.016). The significant risk factors affecting abnormal liver function tests were a maximum ferritin more than 800 ng/mL (*p* = 0.041) and iron chelation (*p* = 0.007). There was no statistically significant difference for the relationship between HCV infection and abnormal liver function. Female gender (*p* = 0.006) and age over 40 years (*p* = 0.003) were significant factors for osteoporosis. No significant risk factors were found in pulmonary hypertension, cholelithiasis, abnormal plasma glucose, and hypothyroidism.

From multivariate analysis, significant risk factors affecting complications in EMH were beta-thalassemia with an odds ratio 5.7 (95% CI 1.2–27.9, *p* = 0.03) and hemoglobin level below 8 g/dL with an odds ratio 7.4 (95% CI 1.7–31.3, *p* = 0.007). Significant risk factors affecting complications in osteoporosis were female gender with an odds ratio 7.4 (95% CI 1.5–38.6, *p* = 0.014) and age more than 40 years with an odds ratio 4.6 (95% CI 1.3–16.5, *p* = 0.017). Iron overload (ferritin > 800 ng/mL) was the only risk factor for abnormal liver function with an odds ratio of 3.7 (95% CI 1.0–13.9, *p* = 0.035) (Table 5).

## 4. Discussion

NTDT is thought to be a less severe form of thalassemia since regular transfusions are not required. However, several studies revealed that many complications occur in patients with this form of thalassemia [8, 11, 14, 19]. We compare prevalence and complications between alpha-NTDT and beta-NTDT and identify putative risk factors affecting complications in this group of patients.

Eighty-three percent of the study population (83 patients) experienced NTDT-related complications. Cholelithiasis (35%), abnormal liver function (29%), and EMH (25%) were the three most common complications found in this study. These results were similar to that found in a study of 37 NTDT patients in Lebanon [6] where common complications were cholelithiasis, pulmonary hypertension, leg ulcer, and EMH. Another study from Taher et al. [8] found that osteoporosis, EMH, hypogonadism, and cholelithiasis were the most common complications in NTDT. These findings indicate that complications from NTDT are quite different from TDT related complications which are mainly cardiomyopathy, endocrinopathy, and abnormal liver function [6].

Differences in the prevalence of complications across NTDT studies can be explained by the various complication definitions used, different in population numbers and type of NTDT (alpha or beta type). Our study had a higher portion of patients with alpha-NTDT which was different from previous studies [6, 8]. This study was done only in adult patients who tended to have more complications.

Another reason that can explain the high prevalence of EMH, cholelithiasis, and iron overload is that our study site is a referral center where most patients within the region with these complications were referred for further treatment.

The lower prevalence of thrombosis in our study may be due to a low incidence of thrombosis for the general Thai population when compared with other countries [31]. Moreover, thrombosis in thalassemia patients was largely disease related such as number of nucleated red cells, platelets ≥ 500 × 10/mm^3^, and splenectomy. The lower number of mean platelet count and splenectomized patients from our study may also explain the lower incidence of thrombosis [8, 32]. Due to incomplete medical records, our study did not have data regarding the number of nucleated red cells and years following splenectomy which are valuable predictors of thrombosis risk.

In this study, we compared the prevalence of complications between alpha-NTDT and beta-NTDT, which contribute new information to the existing body of research focused mainly on beta-NTDT patients [4, 8, 15, 16]. In the multivariate analysis, beta-thalassemia was a significant risk factor for EMH complications (47.8% in beta-NTDT versus 5.6% in alpha-NTDT). However, this significance disappeared when further subgroup analysis into alpha and beta-NTDT populations reduced the number of patients. The alpha-NTDT group tended to have less severe clinical manifestations than the beta group such as the degree of iron overload and iron chelation therapy and splenectomy frequency.

In the OPTIMAL CARE study, splenectomy had a significant effect on almost all complications in TI patients [8] while none of complications in our study related with this condition perhaps due to the low number of splenectomized patients. The rate of splenectomy in our study was 33% compared to more than half in other studies [8].

Iron overload played a significant role in many TI related complications in previous studies and was also a risk factor for abnormal liver function tests in our study [8, 15, 24, 33, 34]. The incidence of iron overload was high in our study where 76% of patients had serum ferritin levels > 800 ng/mL. Half of these patients received iron chelation therapy while the other half could not afford iron chelation therapy. Studies indicate serum ferritin levels may underestimate liver iron burden in NTDT [8], which can explain the high prevalence of iron overload and abnormal liver function in our study. Hence, iron overload is a critical issue for NTDT patients, even ones who are not receiving regular blood transfusions.

The major limitation of this study was the retrospective data collection for some complications such as pulmonary hypertension (PHT), thrombosis, cardiomyopathy, leg ulcers, and osteoporosis (OP). The information was obtained retrospectively from medical records but not routinely accessed, which may cause an underrepresentation of these complications. The incomplete medical records could prevent us from identifying predictive risk factors. Another limitation of this study was the small number of patients comprising the alpha-NTDT and beta-NTDT subgroups.

In conclusion, despite variable clinical presentation and unclear diagnostic criteria, the prevalence of complications in NTDT was higher than and descriptively different from TDT. The prevalence of complications in alpha-NTDT was lower and descriptively different from beta-NTDT.

## Figures and Tables

**Table 1:** Clinical definition required to confirm identified complications.

Complications	Definition
Extramedullary hematopoiesis (EMH)	Physical or radiologic evidence of extramedullary hematopoietic foci with or without symptoms

Pulmonary hypertension (PHT)	Systolic pulmonary artery pressure > 35 mmHg, which corresponds to a tricuspid regurgitant velocity on Doppler echocardiography of > 2.8 m/s plus exertional dyspnea without evidence of left heart disease

Thrombosis	Compression ultrasonography, contrast venography, or angiography evidence of thrombus

Cardiomyopathy	Echocardiographic, electrodiagnostic, or radiologic evidence of pathological change of myocardium such as hypertrophy, dilatation, or restriction

Cholelithiasis	Radiologic evidence of gallbladder stones

Abnormal liver function	ALT > 50 U/L

Pseudoxanthoma elasticum (PXE)	Histopathologic evidence of pathological change in elastic fibers to inelastic tissue

Leg ulcers	Ischemic or necrotic skin lesion on the lower extremity by general visual inspection

Osteoporosis (OP)	Bone densitometry *T* score < 2.5 SD

Abnormal plasma glucose	Fasting plasma glucose > 110 mg/dL at least one time

Hypothyroidism	TSH > 4.7 U/L and a free T4 > 0.8 ng/dL

Hypogonadism	Females: > 13 years, not yet Tanner B2 (i.e., prepubertal breast development) or > 14 years requiring estrogen replacement therapy or > 15 years with primary amenorrhea; males: > 14 years, not yet Tanner G2 (i.e., prepubertal genital development) or on androgen replacement therapy or > 17 years, not yet Tanner G4 (i.e., midpubertal genital development)

Iron overload	Maximum ferritin level >800 ng/mL with or without radiologic or histopathologic evidence

Adapted from [8].

**Table 2:** Characteristics of the study populations.

Parameter	Frequency, number (%)
Gender	
Male	40 (40%)
Female	60 (60%)
Age	
<40 years	54 (54%)
≥40 years	46 (46%)
Region	
Northern Thailand	97 (97%)
Other	3 (3%)
Comorbidities	
Cerebrovascular disease	2 (2%)
Chronic lung disease	4 (4%)
Chronic kidney disease	11 (11%)
Cirrhosis	9 (9%)
Diabetes mellitus	11 (11%)
Dyslipidemia	2 (2%)
Endocrine disease	16 (16%)
Eye-ENT disease	4 (4%)
Gynecologic disease	4 (4%)
Heart disease	8 (8%)
Hypertension	7 (7%)
HBV infection	27 (27%)
HCV infection	12 (12%)
Malignancy	4 (4%)
Seizure	4 (4%)
Personal history	
Alcohol drinking	14 (14%)
Herb use	1 (1%)
Smoking	4 (4%)
Thalassemia type	
Alpha-thalassemia	54 (54%)
Hemoglobin H	38 (38%)
Hemoglobin H/CS	16 (16%)
Beta-thalassemia	46 (46%)
Beta-thalassemia and HbE disease	36 (36%)
Beta-thalassemia intermedia	10 (10%)
Hemoglobin	
Mean hemoglobin level	7.8 g/dL
Platelet	
Mean platelet count	330,900/mm^3^
Serum ferritin, ng/mL	
Mean ferritin	1,563.46 ng/mL
Maximum ferritin > 800 ng/mL	76 (76%)

Hemoglobin H/CS: hemoglobin H with Hb constant spring.

**Table 3:** Treatment, outcome, and complications in study population.

Parameter	Frequency *N* = 100 (%)	*α*-Thal (%) *N* = 54 (%)	*β*-Thal (%) *N* = 46 (%)
Treatment			
Antiplatelet	26%	3 (5.6)	23 (50)
Iron chelation	55%	21 (38.9)	34 (73.9)
Splenectomy	33%	8 (14.8)	25 (54.3)
Complications			
Abnormal plasma glucose	16%	9 (16.6)	7 (15.2)
Abnormal liver function	29%	15 (27.7)	14 (30.4)
Cardiomyopathy	11%	3 (5.5)	8 (17.4)
Cholelithiasis	35%	15 (27.7)	20 (43.5)
Cholecystectomy	25%	10 (18.5)	15 (32.6)
EMH	25%	3 (5.6)	22 (47.8)
Hypothyroidism	13%	6 (11.1)	7 (15.2)
Hypogonadism	7%	2 (3.7)	5 (10.9)
Leg ulcers	2%	1 (1.8)	1 (2.2)
Osteoporosis	17%	11 (20.4)	6 (13.0)
PHT	14%	3 (5.6)	11 (23.9)
PXE	None	None	None
Thrombosis	4%	2 (3.7)	2 (4.3)
Overall complications	83%	43 (79.6)	40 (87)

EMH: extramedullary hematopoiesis, PHT: pulmonary hypertension, PXE: pseudoxanthoma elasticum, *α*-Thal: alpha-thalassemia, and *β*-Thal: beta-thalassemia.

**Table 4:** Significant risk factors affecting complications from univariate analysis.

Complication	Significant variables	*p* value
Extramedullary hematopoiesis	Gender (female)	0.050
	Thalassemia type (beta)	0.031
	Hemoglobin < 8 g/dL	0.003
	Platelets > 400,000 per cumm.	0.025
	Maximum ferritin > 800 ng/mL	0.004
	Splenectomy	0.019
	Iron chelation	0.001

Pulmonary hypertension	None	—

Heart failure	Splenectomy	0.035

Cholelithiasis	None	—

Abnormal LFT (ALT >50 U/L)	Iron chelation	0.007
	Maximum ferritin > 800 ng/mL	0.041
	HCV infection^*∗*^	0.143

Osteoporosis	Gender (female)	0.006
	Age > 40 years	0.003

Abnormal plasma glucose	None	—

Hypothyroidism	None	—

Hypogonadism	Splenectomy	0.016

^*∗*^ *p* value is not significant.

**Table 5:** Significant risk factors affecting complications from multivariate analysis.

Complication	Significant variables	*p* value	95% CI	Odd ratio
Extramedullary hematopoiesis	Thalassemia type (beta)	0.031	1.173–27.971	5.72
	Hemoglobin < 8 g/dL	0.007	1.736–31.252	7.37

Osteoporosis	Gender (female)	0.014	1.514–38.604	7.64
	Age > 40 years	0.017	1.313–16.506	4.66

Abnormal liver function	Maximum ferritin > 800 ng/mL	0.035	1.033–13.919	3.79
